# Examination and Forecast of Relationship among Tourism, Environment, and Economy: A Case Study in Shandong Province, China

**DOI:** 10.3390/ijerph19052581

**Published:** 2022-02-23

**Authors:** Ranran Li, Ziyuan Ding, Yan An

**Affiliations:** 1 School of Management, Shandong Technology and Business University, Yantai 264003, China; liranran1101@163.com; 2 Graduate School of Art & Science, Boston University, Boston, MA 02215, USA; ziyuand@bu.edu; 3 China Institute of Nuclear Industry Strategy, Beijing 100143, China

**Keywords:** tourism industry, ecological environment, the coupling coordination degree

## Abstract

Correctly understanding and handling the relationship of tourism industry, ecological environment, and regional economy is an important prerequisite and foundation for realizing regional ecological protection and high-quality development. Based on the entropy method and the coupling coordination model, this paper conducts quantitative research on the coupling coordination relationship and development law of tourism industry–ecological environment–regional economic (TEE) in various cities in Shandong Province. First, a coupling coordination evaluation system of TEE was constructed to evaluate the comprehensive development level of the three systems in each city in Shandong Province from 2010 to 2017; secondly, based on the coupling coordination model, the relationship among the three systems of each city was analyzed using spatial and temporal dimensions; finally, the gray GM (1, 1) model was used to predict the future coupling coordination degree of the three systems in Shandong Province. The research results show that: (1) the development of the economy and tourism industry of cities in Shandong Province is highly correlated, and the overall trend is increasing. The ecological environment mainly changes first, and then rises. (2) From the perspective of time, the changes in the coupling coordination degree of the three systems are mainly to maintain stability and increase fluctuations, and generally develop in the direction of benign coordination. From a spatial perspective, the coupling coordination degree of the three systems shows significant regional integrity and differences, showing a pattern of high in the east and low in the west. (3) In the next few years, the coupling coordination degree of the three systems will roughly continue the characteristics of changes from 2010 to 2017.

## 1. Introduction

Since the beginning of the 21st century, tourism has become one of the strongest and most dynamic industries in global economic activities. In 2012, China became the world’s third-largest inbound tourism reception country and outbound tourism consumption country, and the scale of the domestic tourism market ranks first in the world, with nearly 3 billion visits. According to the “China Tourism Development Report 2016”, China has formed the world’s largest domestic tourism market. As one of the national strategic pillar industries, tourism has played a significant role in driving the development of related industries, creating job opportunities, and increasing tax revenues [1]. As of the end of 2017, the overall contribution of tourism to the national economy and the ratio of tourism employment to the national employment population have reached more than 10%. At present, 30 provinces across the country have identified tourism as a strategic pillar industry or advantageous leading industry [2]. However, while the development of tourism and economy brings huge economic and social benefits, it may also cause damage to the natural environment. After the environmental degradation exceeds its threshold, it will increase the cost of economic activities and restrict the long-term development of regional tourism [3,4]. Therefore, ways in which to coordinate the relationship between tourism resource development, ecological environment protection, and regional economic development have become important topics that the academic circles of various countries are generally concerned about, and that local governments are working hard to resolve.

Domestic and foreign scholars have conducted a lot of research on the relationships between the tourism industry, the ecological environment, and the regional economy. Most of them focus on the analysis of the relationship between tourism and environment, tourism and economy, and economy and environment. Between tourism and the environment, Wall et al., earlier elaborated on the impact of tourism activities on the ecological environment, and analyzed the interaction mechanism between the two [5]. Gossling conducted in-depth discussions on environmental issues such as land cover, energy use, biological exchange, and extinction of wild species that may be brought about by tourism activities [6]. Day et al., specifically analyzed the challenges of the sustainable development of regional tourism in terms of energy and environment in the United States and China [7]. Zhang et al., conducted an in-depth study on the coupling degree of tourism and ecology in China’s coastal regions based on weighted principal components and technique for order preference by similarity to an ideal solution [8]. Tang introduced an index system and developed an integrated approach to evaluate the coupling coordination between tourism and the environment [9], and found that tourism has a positive impact on Malaysia’s economic growth both in the short-run and in the long-run [10].

In the research of the relationship between tourism and economy, Wu et al., explored the causal relationship between international tourism receipts and economic growth in China’s 11 eastern provinces, accounting for both dependency and heterogeneity across provinces [11]. Ahmad explored the nexus between tourism and environmental pollution for three lower middle-income Southeast Asian economies: Indonesia, the Philippines, and Vietnam [12]. Law et al., framed the green economy concept from a tourism perspective, and presented a model for translating the green economy concept into a tourism stakeholder engagement process [13]. KC et al., explored the Nepalese tourism industry’s role in addressing the United Nations Sustainable Development Goals (SDGs) from the perspective of various tourism stakeholders. The findings suggest that, to varying degrees, the SDGs are applicable as well as achievable for Nepal [14]. Chidakel et al., proposed economic impacts from tourism may benefit people living near parks and contribute to national-level economic growth [15].

In the research of the relationship between economy and environment, Norgaard proposed that the economy and the environment could be coordinated through a feedback loop [16]. Hanleya et al., quantitatively evaluated the coordinated development of Scotland’s economy and environment from 1980 to 1993 [17]. Dinda found that the coordination degree between environmental quality and economic income may be an “N” type development trend [18]. Zhang et al., offered a complex evaluation index system for Heilongjiang tourism, urbanization, and ecological environmental development based on coupling mechanisms across these three subsystems [19]. By choosing the 14 cities in Gansu Province as cases, Lu et al., quantified the economy–environment interactions in tourism with a combination of varying quantitative methods including order parameter analysis, fuzzy membership classification, regression analysis, and gray correlation analysis measurement models [20].

From extensive research and practice, the tourism industry-ecological environment-regional economy (TEE) can be regarded as an open system with extensive content, with complex structures and coupling characteristics and a deep understanding of the interaction mechanism among the three systems is the primary problem for their coordinated development. First of all, tourism has become a key link for coordinating regional economy and ecological environment due to its strong economic driving force and low direct pollution [21]. While tourism promotes the development of regional catering, accommodation, entertainment, transportation, and telecommunications, etc., it can also enhance the flow of material, information, and personnel between the region and the outside world, thereby increasing the degree of openness of the regional economy. Through the development of eco-tourism, science, and education tourism, etc., the natural environment can be protected and rationally utilized. Secondly, the ecological environment is the material basis of economic activities, and an important guarantee for the sustainable development of tourism. On the one hand, the natural ecology provides abundant natural resources and energy power for economic development; on the other hand, the ecological environment is also a valuable ecological resource for the tourism industry, and it is a prerequisite for the survival and development of the tourism industry. Finally, the regional economy plays an important role in promoting environmental protection and tourism development. Economic development provides certain financial guarantees and technical support for environmental protection and ecological optimization, relying on the transformation of production methods to reduce energy consumption and reducing ecological pressure; at the same time, economic development provides more complete infrastructure and service facilities for the tourism industry, and promotes the development and upgrade of regional tourism [22]. In short, the tourism industry is the key to coordination, the ecological environment is the foundation for development, and the social economy is an important support. If any of these aspects is ignored, the entire regional community development system will fall into imbalance and chaos [23].

However, the existing literature still focuses on the analysis of the relationship between the tourism industry, the ecological environment, or the regional economy, and there are relatively few systematic studies that unify the three organically [24,25,26]. Research units mostly focus on single cities [27,28] or special types of tourism destinations such as islands [29,30], and comparative analysis based on macro scales is less. In addition, the research period focuses on cross-sectional data or the grasp of short-term development periods, and lacks prediction of the future coordination of the three systems [31,32]. Shandong Province is a major economic province on the eastern coast of China. It has certain advantages in terms of location, transportation, resource endowments, industrial foundation, and talent reserves. However, it also has problems such as an unreasonable economic structure, insufficient development potential, high total energy consumption, and large total discharge of pollutants, which call for an urgent need to adjust the economic structure and change the development mode. As an industry with strong radiation and relevance, the tourism industry is an important driving force for the adjustment of regional economic structure. In the construction of the country’s first comprehensive experimental zone for the conversion of new and old kinetic energy, the tourism industry, one of the top ten industries to be developed in Shandong Province, is facing new development opportunities and challenges. At the same time, behind the rapid development of the tourism industry and regional economy, there are also serious environmental hidden dangers. In addition, the economic scale and industrial structure of each city are quite different, and the degree of impact on the ecological environment and the degree of protection are also different. Using scientific methods to quantify and analyze the coupling coordination relationship of TEE among the cities in Shandong Province are of great significance to the realization of the high-quality development of the overall economy in Shandong Province.

Based on this, the article builds a coupling coordination evaluation index system of TEE, taking 17 cities in Shandong Province as the research object, based on the coupling coordination degree model to study the relationship of the three systems quantitatively from the two dimensions of time and space, explore the problems that affect the coordinated development of the three systems. This article also uses the gray GM (1, 1) model to predict the coordinated development of Shandong Province in the next few years, in order to provide some suggestions for the coordinated development of TEE in Shandong Province and various cities, which also can provide reference for the coordinated development of the three systems of other provinces and cities.

## 2. Materials and Methods

### 2.1. Study Area

Shandong Province is located between 34°22.9′–38°24.01′ north latitude and 114°47.5′–122°42.3′ east longitude along the eastern coast of China, bordering Hebei, Henan, Anhui, and Jiangsu provinces from north to south. Shandong covers an area 721.03 km long from east to west, and 437.28 km long from north to south, and the province has a land area of 155,800 square kilometers. There are various types of landforms, including mountains, hills, platforms, basins, plains, lakes, etc.; it spans the five major river systems of Huaihe, Yellow, Haihe, Xiaoqinghe, and Jiaodong; it belongs to a warm temperate monsoon climate. Shandong Province has jurisdiction over 17 prefecture-level cities. In January 2019, Laiwu City was merged into Jinan City, thus this became 16 prefecture-level cities. As of the end of 2019, the resident population of Shandong Province was 100.7021 million. In 2019, the total tourism revenue of Shandong Province was 1108.73 billion yuan, an increase of 12.1% over the previous year; 938.093 million domestic and foreign tourists were received, an increase of 8.6%.

### 2.2. Data Source and Data Pre-Processing

The relevant statistics were derived from the Shandong Statistical Yearbook (2010–2017), the Statistical Bulletin of National Economic and Social Development of Shandong Province (2010–2017), the China Tourism Statistical Yearbook (2010–2017), the China Tourism Yearbook (2010–2017), the China City Statistical Yearbook (2010–2017), the China Statistical Yearbook (2010–2017), the China Regional Economic Statistics Yearbook (2010–2017), and the China City Construction Statistical Yearbook (2010–2017), etc., as well as the Shandong Provincial Statistics Information Network, the Shandong Provincial Tourism Bureau, the Environmental Protection Bureau, and other government authoritative websites. As the dimensions, magnitude, and positive and negative orientations of the raw data of the selected indicator are different, the data should be standardized. All indicators can be divided into positive and negative classes. If the positive indicator is large, then the conditions for system development would be favorable (conversely, the conditions are favorable if the negative indicator is great). The standardization process was shown in Equations (1) and (2).

(1) $$Positive\;indicator:\;X_{ij}^{\prime }=\frac{X_{ij}-X_{minj}}{X_{maxj}-X_{minj}}$$

(2) $$Negative\;indicator:\;X_{ij}^{\prime }=\frac{X_{maxj}-X_{ij}}{X_{maxj}-X_{minj}}$$

where $X_{ij}$ represents the value of indicator j in year i, and $X_{maxj}$ and $X_{minj}$ indicated the maximum and minimum values, respectively, of indicator j among all years. All exponentialvalues after processing are within the range of [0, 1].

### 2.3. Methods

#### 2.3.1. The Indexes for Evaluation of Tourism, Environment, and Economy

In order to highlight the important coordination and linking role of the tourism industry in the regional economy and ecological environment, the three subsystems of tourism industry, ecological environment, and regional economy are placed at the same level for research. At the same time, considering that the tourism industry is an important part of economic activities, the analysis needs to focus on the affiliation and weight of the three. An evaluation system with comprehensive content and reasonable levels is the prerequisite basis for the coupling and coordination analysis among the three. Based on the principles of data availability, indicator representativeness, and system relevance, and with reference to relevant research results [23,33,34], centering on the three cores of tourism, environment and economy, starting from the eight dimensions of tourism economic benefit, tourism market scale, tourism industry level, ecological environment resources, ecological environment pollution, ecological environment governance, total economic scale, and economic structure characteristics, 28 one-way indicators are selected in detail, and indicators are taken into consideration. Taking into account the horizontal and vertical matching and comparability of the indicators, the evaluation system for the coordinated development of TEE is established (as shown in Table 1).

#### 2.3.2. The Entropy Method

In order to determine the weight of each indicator in the tourism, environment, and economy index systems, the entropy method was employed [35]. The weight of each indicator was calculated according to information entropy and variations in the indicators. The detailed steps for calculating the weight of each indicator were as follows (where *n* is the number of indicators, and *m* denotes years):

The proportion of the indicator *j* in year *i*:

(3) $$r_{ij}=X_{ij}^{\prime }/\overset{m}{\underset{i=1}{\sum }}X_{ij}^{\prime }$$

Information entropy of the indicator:

(4) $$H_{j}=-\frac{1}{\ln m}\;\overset{m}{\underset{i=1}{\sum }}r_{ij}$$

Entropy redundancy:

(5) $$f_{j}=1-H_{j}$$

Weight of the indicator:

(6) $$w_{j}=f_{j}/\overset{n}{\underset{j}{\sum }}f_{j}$$

#### 2.3.3. The Coupling Coordination Degree Model

Coupling is a phenomenon whose concept is derived from physics. It refers to the process of two or more complex systems interacting and affecting through various subsystems or elements to promote the process of the system from disorder to order, and determines the phase change of the system [35]. Coupling degree is used to measure the degree of interaction between various systems. Coupling coordination degree is a measure of the degree of consistency of coordination between various systems in different regions during the same period. The coupling coordination model has been widely used in the coordinated development of different systems such as urbanization, ecological environmental protection, transportation accessibility, and tourism economy. It is an important model for studying the coordinated development of regional economy, industry, transportation, and ecological environment.

Based on the evaluation system for the coordinated development of the TEE constructed previously, the coupling coordination degree model can be used to analyze the coordination status of tourism, environment, and economy in different cities in different development periods quantitatively. The comprehensive benefit evaluation functions of the three subsystems of tourism, environment and economy are as follows:

(7) $$F\left(x\right)=\overset{m}{\underset{i=1}{\sum }}a_{i}x_{i}^{\prime }$$

(8) $$G\left(y\right)=\overset{n}{\underset{i=1}{\sum }}b_{i}y_{i}^{\prime }$$

(9) $$H\left(x\right)=\overset{k}{\underset{i=1}{\sum }}c_{i}z_{i}^{\prime }$$

where *F(x), G(y), H(z)* represent the comprehensive benefits of the tourism subsystem, environmental subsystem, and economic subsystem, respectively; $a_{i}$, $b_{i}$, $c_{i}$ are the weights of the indicators in each subsystem (that is, calculated by entropy weight method in Section 2.3.2). $x_{i}^{\prime },\;\;y_{i}^{\prime },\;z_{i}^{\prime }$ are dimensionless index values of the tourism subsystem, environmental subsystem, and economic subsystem respectively.

To calculate the coupling degree of the three systems, with the aid of the coupling coordination model in physics, the formula for coupling degree of TEE three systems is obtained.

(10) $$C={\left\{\frac{F\left(x\right)\times G\left(y\right)\times H\left(z\right)}{{\left[\frac{F\left(x\right)+G\left(y\right)+H\left(z\right)}{3}\right]}^{3}}\right\}}^{\frac{1}{3}}$$

where *C* is the coupling degree. When *C* = 1, it indicates that the three systems are in the best coupling state; when *C* = 0, it indicates that the internal elements of the system are irrelevant, and the system develops disorderly.

When calculating the degree of coupling and coordination of the three systems, it should be noted that coupling can only indicate the degree of interaction between the various systems in the study area, but cannot reflect the level of coupling and coordination in different regions at the same time, thus the coupling coordination degree model is introduced to determine the coordinated development degree of the three systems.

(11) $$D=\sqrt{C\times T},T=\alpha F\left(x\right)+\beta G\left(y\right)+\gamma H\left(z\right)$$

where *C* is the coupling degree; *D* is the degree of coupling coordination, $D\in \left(0,1\right)$ (the greater the value of *D* close to 1, the better the coupling effect of the system); *T* is the comprehensive evaluation index of the three systems; and α,  β,  γ are undetermined coefficients. Shandong Province locates on the eastern coast, with developed social economy and tourism. Industrialization and urbanization are advancing rapidly, and environmental pollution and ecological destruction have restricted the sustainable and healthy development of the economy and society. Therefore, economic development and environmental protection are equally important. Economic development is the result of the combined effects of the three industries, and tourism is only a part of the tertiary industry. Therefore, α=0.2,β=0.4, and γ=0.4 are taken. The distribution function [36] is used to determine the division standard of coupling coordination degree, which is shown in Table 2.

#### 2.3.4. Grey Forecasting Method of GM (1, 1)

Gray forecasting model is a forecasting model established for systems with uncertain levels and structures, and dynamic changes with random characteristics, also known as the GM model. By accumulating and averaging the original random variables, a differential equation model is constructed to make predictions for future data [37,38,39,40].

The modeling steps of the GM (1, 1) model are

(1) Assume that $X^{\left(0\right)}$ is a non-negative data sequence

(12) $$X^{\left(0\right)}=[X^{\left(0\right)}(1),X^{\left(0\right)}(2),\cdots ,X^{\left(0\right)}(n)]$$

where $X^{\left(0\right)}\left(k\right)$ is the *k*-th value of the sequence $X^{\left(0\right)}$ (*k* = 1,2,…,*n*).

(2) The first-order cumulative sequence $X^{\left(1\right)}$ is

(13) $$X^{\left(1\right)}=[X^{\left(1\right)}(1),X^{\left(1\right)}(2),\cdots ,X^{\left(1\right)}(n)]$$

where $X^{\left(1\right)}(i)=\overset{i}{\underset{k=1}{\sum }}X^{\left(0\right)}(k),\;i=1,2,\cdots ,n$.

(3) If $X^{\left(0\right)}$ and $X^{\left(1\right)}$ satisfy a series of tests, then the $X^{\left(1\right)}$ sequence has an exponential growth law, that is, it satisfies the first-order linear differential equation:

(14) $$\frac{dX^{\left(1\right)}}{dt}+aX^{\left(1\right)}=u$$

In the formula, *a* is the development gray number, which reflects the development trend of $X^{\left(0\right)}$ and $X^{\left(1\right)}$; *u* is called the endogenous control gray number, which reflects the change relationship between data.

(4) In order to solve the parameter *a* and *u*, let $\hat{a}={\left(a,u\right)}^{T}$ be the vector to be evaluated, discretize the formula (14), and use the least square method to approximate it as:

(15) $$\hat{a}={\left(B^{T}B\right)}^{-1}B^{T}Y_{n}$$

where:

$$B=\left[\begin{array}{cc} -Z^{\left(1\right)}(2) & 1\\ -Z^{\left(1\right)}(3) & 1\\ \vdots & \vdots \\ -Z^{\left(1\right)}(n) & 1 \end{array}\right]=\left[\begin{array}{cc} -\frac{1}{2}[X^{\left(1\right)}(1)+X^{\left(1\right)}(2)] & 1\\ -\frac{1}{2}[X^{\left(1\right)}(2)+X^{\left(1\right)}(3)] & 1\\ \vdots & \vdots \\ -\frac{1}{2}[X^{\left(1\right)}(n-1)+X^{\left(1\right)}(n)] & 1 \end{array}\right],\;Y_{n}=\left[\begin{array}{c} X^{\left(0\right)}(2)\\ X^{\left(0\right)}(3)\\ \vdots \\ X^{\left(0\right)}(n) \end{array}\right].$$

(5) After obtaining *a* and *u*, continue to solve the differential Equation (14) and discretize to obtain:

(16) $${\hat{X}}^{\left(1\right)}\left(k+1\right)={ce}^{-ak}+\frac{u}{a},k=0,1\cdots ,n-1.$$

Assuming that the initial conditions are ${\hat{X}}^{\left(1\right)}\left(1\right)=X^{\left(0\right)}\left(1\right)$, then the solution of Equation (14) is:

(17) $${\hat{X}}^{\left(1\right)}\left(k+1\right)=\left[X^{\left(0\right)}(1)-\frac{u}{a}\right]e^{-ak}+\frac{u}{a},k=0,1,\cdots ,n-1.$$

(6) Accumulate and subtract formula (17) to obtain the gray prediction model of the original series $X^{\left(0\right)}$:

(18) $${\hat{X}}^{\left(0\right)}\left(k\right)={\hat{X}}^{\left(1\right)}\left(k\right)-{\hat{X}}^{\left(1\right)}\left(k-1\right),k=2,3,\cdots ,n.$$

The accuracy test of the gray prediction formula is generally given in Table 3. If both the *p*-value and *C*-value are within the allowable range, the predicted value of this indicator can be calculated. Otherwise, the formula needs to be revised again by analyzing the residual sequence.

## 3. Results and Discussion

### 3.1. Evaluation of the Development of Each Subsystem

**Figure 1:**
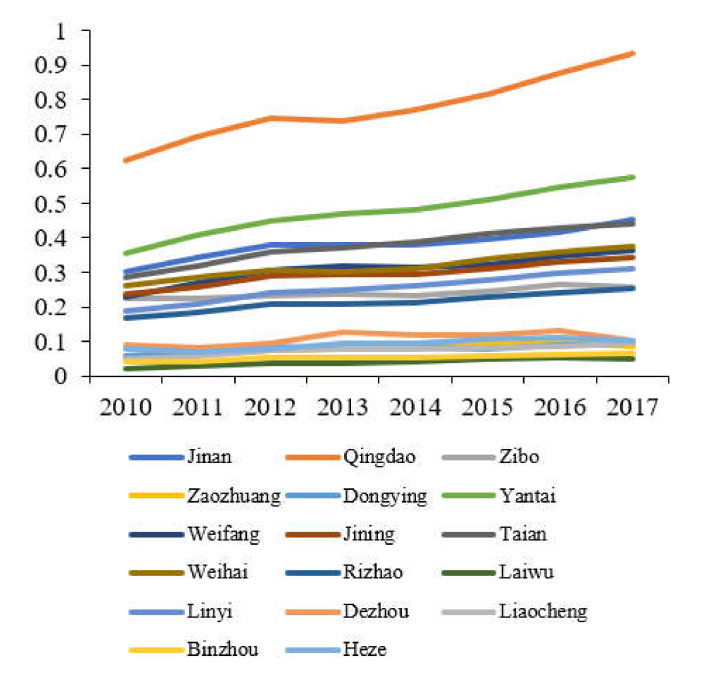
Evaluation value of the tourism systems of cities in Shandong Province.

**Figure 2:**
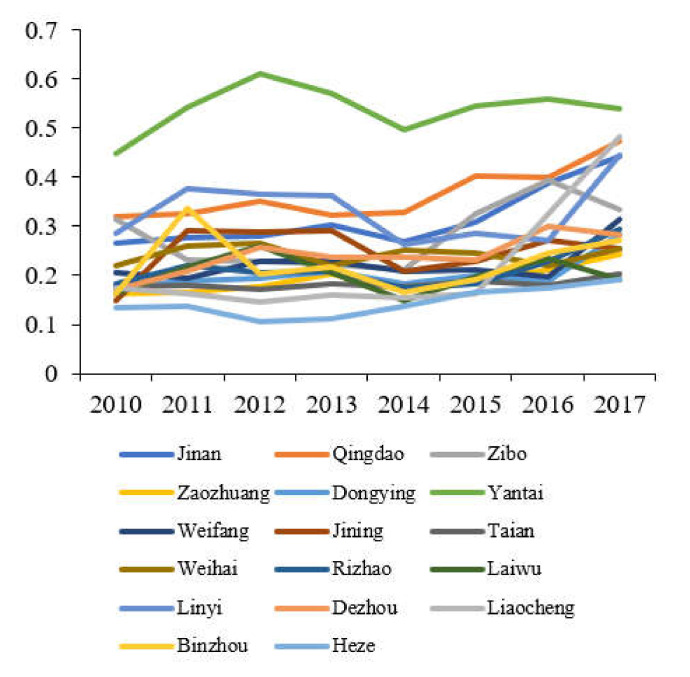
Evaluation value of the ecosystems of cities in Shandong Province.

**Figure 3:**
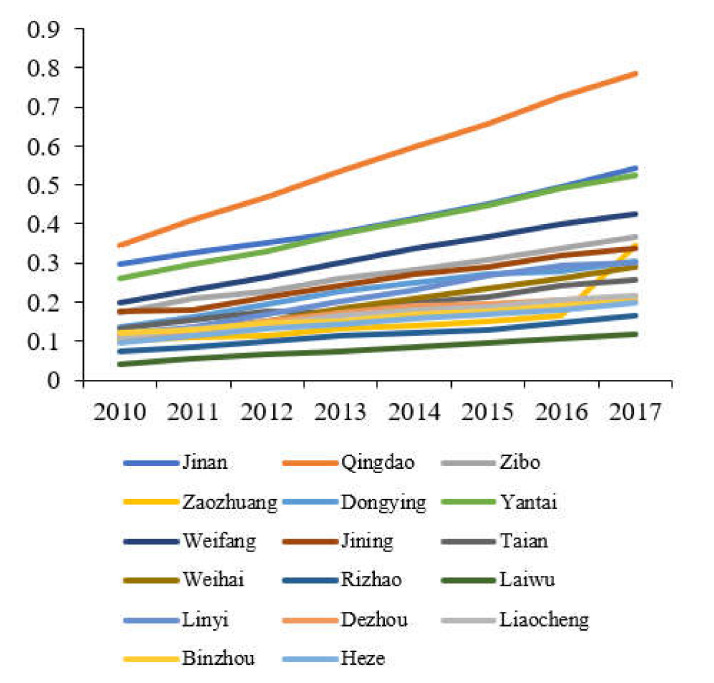
Evaluation value of the economic systems of cities in Shandong Province.

The comprehensive evaluation value of each subsystem of tourism, environment, and economy in Shandong Province is calculated according to Equations (7)–(9), and a line graph of the comprehensive benefit of tourism, economy and environment in Shandong Province is produced, as shown in Figure 1, Figure 2 and Figure 3.

The overall tourism development of 17 cities in Shandong Province is on the rise, but there are obvious regional differences. These differences are due to the geographical location, socio-economic conditions, public service levels, transportation accessibility, tourism resource endowment, tourism development capital investment, and operation and management level, etc. According to the difference in the level of tourism development between regions in Shandong Province represented by the benefit evaluation value of the tourism subsystem, the 17 cities in Shandong Province can be divided into three development gradients. The first gradient is Qingdao City. The tourism development of Qingdao is far ahead of other cities. During the study period, the tourism benefit evaluation value has been maintained at a level above 0.6, and it is on an upward trend. In 2017, it was higher than 0.9. The second gradient shows the cities of Yantai, Jinan, Taian, Weihai, Jining, Weifang, Zibo, Linyi, and Rizhao. From 2010 to 2017, the comprehensive tourism benefits of these nine cities show an overall upward trend at the range of 0.15–0.6, and the rising speed is different due to different development conditions. Among them, Yantai City’s tourism development level has relatively high, second only to Qingdao City, and its tourism benefit evaluation value remained at 0.35–0.6 during 2010–2017. The remaining seven cities of Dezhou, Heze, Dongying, Zaozhuang, Liaocheng, Binzhou, and Laiwu are in the third gradient. From 2010 to 2017, the comprehensive tourism benefits of these cities have been hovering at a low level, and the tourism evaluation value is below 0.1, the development level of which is much lower than the first two gradient cities.

The data in Figure 2 shows that the eco-environmental evaluation values of cities in Shandong Province mainly fall and then rise, and most of them have inflection points in 2014. The development of its ecological environment conforms to the inverted “U”-shaped environmental Kuznets curve. The ecological environment was damaged by economic growth in the previous period. However, as the regional economic structure adjustment and environmental protection increased, the ecological environment evaluation value began to fluctuate and rise. Among them, the environmental benefit evaluation value of Yantai has always been at the leading level. Since 2011, it has basically remained above 0.5. The environmental benefit evaluation values of other cities are all at a relatively low level. Except for the four cities of Liaocheng, Qingdao, Linyi, and Jinan in 2017, the rest of the environmental benefit evaluation values are all below 0.4, especially in Heze, whose environmental benefit evaluation value has been below 0.2, and the ecological environment problem is relatively serious.

As shown in Figure 3, the economic benefits of 17 cities in Shandong Province are increasing year on year. Qingdao is in the leading position, followed by Jinan and Yantai, then followed by Weifang, Zibo, Jining, Dongying, Linyi, Weihai, Tai’an, Dezhou, Liaocheng, Binzhou, Heze, Zaozhuang, Rizhao, Laiwu.

From the comparison of Figure 1, Figure 2 and Figure 3, the development of the two systems of economy and tourism industry in Shandong Province has strong similarities. The top three are Qingdao, Jinan, and Yantai, and their changes are the same. In addition, at the bottom of the two major systems are Laiwu, which indicates that the regional economy and the development of the tourism industry have a high correlation. The economic development supports and promotes the tourism industry, and the weak economic foundation will restrict the long-term development of the regional tourism industry. The change characteristics of the ecological environment system of 17 cities in Shandong Province are different from the economic and tourism systems. The highest ecological environment evaluation value is Yantai, and the lowest city is Heze. This shows that the ecological environment is incompatible with tourism and economy. There is no significant correlation, and there is no conflict between economic development and ecological protection. While the development of regional economy and tourism industry, the optimization of the ecological environment can be promoted by advocating ecological civilization and developing circular economy.

**Figure 4:**
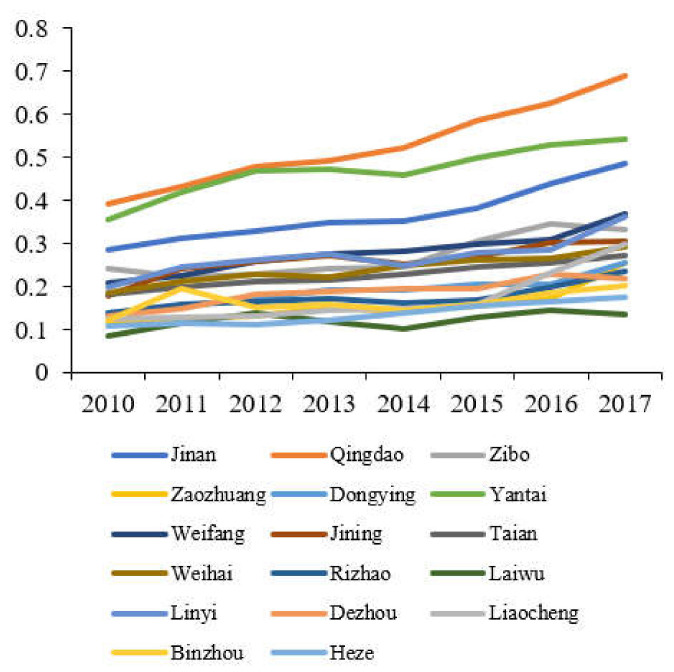
Comprehensive development indexes of the three systems of cities in Shandong Province.

According to the coupling coordination model, the comprehensive evaluation index of the three systems can be calculated to reflect the difference in the comprehensive development of TEE in 17 cities in Shandong Province. The results are calculated according to Equation (10), and shown in Figure 4. According to the change trend of the comprehensive evaluation index of the three systems in Shandong Province from 2010 to 2017, Qingdao, Yantai and Jinan are the top three, and the comprehensive evaluation indexes of the remaining 14 cities are all below 0.35. It can be seen from this that the overall development level of TEE in Shandong Province is still not ideal. It needs to be driven by the three core cities of Qingdao, Yantai, and Jinan to continue to promote the comprehensive development of the three systems.

### 3.2. Analysis on the Spatio-Temporal Evolution of Coupling Coordination Degree

**Figure 5:**
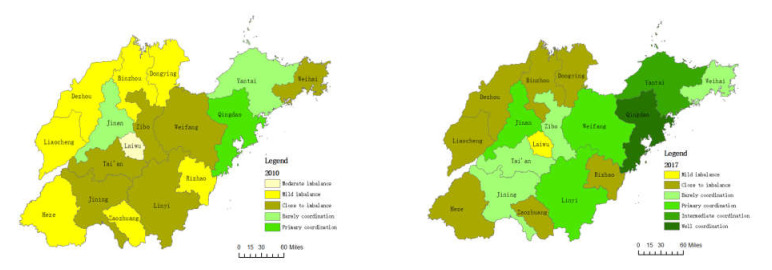
Coupling coordinative type of TEE of Shangdong Province in 2010 and 2017.

The evaluation values of the tourism industry, ecological environment, and regional economic subsystems of each city in Shandong Province were respectively entered into the coupling coordination degree index formulas (Equation (11)) to obtain the coupling coordination degree index of the three systems of TEE (as shown in Table 4). Combining the classification and evaluation standards of the coupling coordination degree of the three systems, and for the convenience of research, the two years of 2010 and 2017 were selected for use in GIS to deeply explore the characteristics of its temporal and spatial evolution (as shown in Figure 5).

From the perspective of time evolution, the coupling coordination degree of TEE of 17 cities in Shandong Province mainly remained stable and fluctuated upward from 2010 to 2017, and generally developed towards a benign coordinated direction. Among them, the degree of coupling coordination among Qingdao, Yantai, Weifang, and Linyi increased by two levels. Specifically, Qingdao went from primary coordination to well coordinated, Yantai went from barely coordinated to intermediate coordination, and both Weifang and Linyi went from close to imbalance to primary coordination. All other cities have increased by one coupling interval. It can be seen that after years of development, various cities have made certain progress in the coordinated development of the three systems, but there are still seven cities in the marginal range of imbalance, especially Laiwu, which has the lowest degree of coupling coordination, is in a mild imbalance. Therefore, it is still necessary to take comprehensive measures to improve the coordination of the development of the tourism industry, the ecological environment, and the regional economic system, and prevent the regional social economic system from falling into imbalance and continuously deteriorating.

From a spatial perspective, the coupling coordination degree of TEE in 17 cities in Shandong Province shows significant regional integrity and differences. With the exception of several abnormally changing cities such as Jinan, Laiwu, Linyi, Rizhao, and Weihai, the coordination level of TEE coupling in Shandong Province is gradually increasing from west to east. Due to the different impacts of the tourism industry, social and economic development level, environmental protection investment, environmental policy, and regional development strategy on the ecological environment in each city, there are obvious regional differences in the level of coupling coordination.

### 3.3. Forecast of TEE Coupling and Coordinated Development

Based on the gray GM (1, 1) prediction model, the coupling coordination degrees of the three systems in 17 cities in Shandong Province from 2010 to 2017 are taken as analysis data to predict the coupling coordination degrees of the three systems over the next five years. The results are calculated according to Equations (17) and (18) and shown in Table 5. Among them, in January 2019, Laiwu City merged into Jinan City, thus only 2018 forecasts are made for these two cities. The data in Table 5 shows that the development of the coupling coordination degree of TEE in Shandong Province in the next few years will roughly continue the change characteristics from 2010 to 2017, and all cities will show a slight upward development trend. Among them, the coupling coordination degree of the three systems in Qingdao will increase from well coordinated to high-quality coordination, Yantai will upgrade from intermediate coordination to good coordination, and Zibo will upgrade from primary coordination to intermediate coordination. Weifang, Jining, Weihai, and Liaocheng will go from barely coordinated to primary coordination, and Zaozhuang, Dongying, Rizhao, Dezhou, and Heze will be from close to imbalance to barely coordinated. The forecast results show that although the coordinated development of the TEE in Shandong Province will improve in the next few years, its coupling level will increase and evolve slowly. With the exception of Qingdao, it will take a long time to achieve the mutual promotion and coordinated development of the three systems in each city, which requires cities in the future development to focus on breaking through their own leading restrictive factors, to achieve the coordinated synchronization and overall improvement of economic restructuring, ecological environmental protection, and tourism industry development.

## 4. Conclusions

This article analyzes the coupled and coordinated development trend of TEE in 17 cities in Shandong Province by establishing an evaluation index system and using a coupling coordination degree model. The conclusions are as follows:

The development of the economy and tourism industry of 17 cities in Shandong Province is highly correlated. Qingdao, Jinan, and Yantai are all ranked in the top three, and the trend of their changes are the same. With favorable policy guidance and complete supporting facilities, the tourism industry is more flexible and competitive than regional macroeconomics.

The ecological environment mainly falls first, and then rises. There is no significant conflict between environmental protection and economic development. While regional economy and tourism are developing, the ecological environment can be improved by advocating ecological civilization and developing circular economy.

From the perspective of time evolution, the coordination degree of TEE coupling of 17 cities in Shandong mainly remained stable and fluctuated from 2010 to 2017. However, individual cities have also experienced a slight decline at a certain period of time, and ecological protection needs to be emphasized at all times. From a spatial point of view, the coupling coordination degree of the three systems in Shandong Province is high in the east and low in the west.

The coupling coordination degree of TEE in 17 cities in Shandong Province will generally continue the characteristics of changes from 2010 to 2017 in the next few years. The forecast results show that the coordinated development of Shandong’s TEE in the next few years will generally improve. However, the pace of upgrading and evolution is relatively slow, and each city needs to break through the weak links according to its own conditions, and then realize the coordinated development of the three systems in Shandong Province.

This research analysis the coupling coordination level of TEE based on cities in Shandong Province, breaks through the limitations of the single city as the analysis unit and the two–two coupling relationship as the research content. The prediction provides a basis for decision-making for regional economic restructuring, ecological environmental protection, and the development of the tourism industry. However, due to the limitations of data acquisition, the established coupling coordination evaluation system may not fully reflect the development of the three systems in each city. The greater randomness of some original sequences in the gray prediction model will also have a certain impact on the final prediction accuracy. Further optimization in this area will be researched. Furthermore, the impact of the COVID-19 was not considered in the forecast, therefore this complex impact in prediction will also be researched in the future.

## Figures and Tables

**Table 1:** Evaluation index system for the coordinated development of TEE.

Systems	Primary Indicators	Secondary Indicators	Direction
Tourism Economic System	Tourism economic benefit	Domestic tourism revenue (hundred million yuan)	+
		Foreign exchange revenue from international tourism (10,000 USD)	+
	Tourism market scale	Number of domestic tourists (10,000 people)	+
		Number of inbound tourists (10,000 people)	+
	Tourism industry level	Proportion of total tourism revenue in GDP (%)	+
		Proportion of total tourism revenue in the tertiary industry (%)	+
		Number of employees in accommodation and catering services above designated size (people)	+
		Business revenue of accommodation and catering above designated size (10,000 yuan)	+
Ecological environment system	Ecological environment resources	Total afforestation area (m^2^)	+
		Park green area per capita (m^2^/people)	+
		Water resources per capita (m^3^/people)	+
	Ecological environment pollution	Wastewater discharge (10,000 t)	−
		Sulfur dioxide emissions (t)	−
		Smoke and dust emissions (t)	−
		Industrial solid waste production volume (10,000 t)	−
	Ecological environment governance	Domestic garbage removal volume (10,000 t)	+
		Total sewage treatment (10,000 t)	+
		Comprehensive utilization of industrial solid waste (10,000 t)	+
		Industrial solid waste disposal volume (10,000 t)	+
Regional economic system	Total economic scale	GDP (hundred million yuan)	+
		GDP per capita (yuan/people)	+
		Added value of tertiary industry (hundred million yuan)	+
		Proportion of tertiary industry in GDP (%)	+
		Total retail sales of social consumer goods (10,000 yuan)	+
		Fixed asset investment in the whole society (hundred million yuan)	+
	Economic structure characteristics	Per capita disposable income of urban households (yuan)	+
		General Public Budget Revenue (10,000 yuan)	+
		Urban registered unemployment rate (%)	−

**Table 2:** The classification criteria of coupling coordinative degree.

Coupling Coordinative Degree	Coordination Level	Coupling Coordinative Degree	Coordination Level
0.00–0.09	Extreme imbalance	0.50–0.59	Barely coordinated
0.10–0.19	Severe imbalance	0.60–0.69	Primary coordination
0.20–0.29	Moderate imbalance	0.70–0.79	Intermediate coordination
0.30–0.39	Mild imbalance	0.80–0.89	Well coordinated
0.40–0.49	Close to imbalance	0.90–1.00	High quality coordination

**Table 3:** The accuracy test grade of gray forecast model.

Accuracy Grade	*p*	*C*	Accuracy Grade	*p*	*C*
good	>0.95	<0.35	inadequate	>0.70	<0.65
qualified	>0.80	<0.5	unqualified	≤0.70	≥0.65

**Table 4:** Coupling coordination degree of TEE of cities in Shandong Province.

Cities	2010	2011	2012	2013	2014	2015	2016	2017
Jinan	0.53	0.56	0.57	0.59	0.59	0.61	0.66	0.69
Qingdao	0.61	0.64	0.67	0.68	0.7	0.75	0.77	0.81
Zibo	0.48	0.47	0.48	0.49	0.49	0.55	0.58	0.57
Zaozhuang	0.33	0.34	0.35	0.37	0.37	0.38	0.4	0.47
Dongying	0.35	0.38	0.4	0.41	0.42	0.42	0.43	0.47
Yantai	0.59	0.64	0.67	0.68	0.68	0.71	0.73	0.73
Weifang	0.46	0.47	0.51	0.52	0.52	0.54	0.54	0.6
Jining	0.42	0.49	0.51	0.52	0.5	0.52	0.55	0.55
Taian	0.41	0.43	0.44	0.45	0.46	0.48	0.49	0.51
Weihai	0.42	0.45	0.47	0.47	0.49	0.51	0.51	0.54
Rizhao	0.36	0.38	0.39	0.41	0.4	0.41	0.44	0.48
Laiwu	0.24	0.28	0.31	0.31	0.3	0.33	0.35	0.34
Linyi	0.43	0.47	0.5	0.52	0.5	0.53	0.53	0.6
Dezhou	0.36	0.37	0.41	0.43	0.43	0.43	0.46	0.45
Liaocheng	0.33	0.34	0.35	0.37	0.37	0.38	0.45	0.49
Binzhou	0.32	0.37	0.36	0.37	0.36	0.37	0.4	0.42
Heze	0.32	0.33	0.33	0.35	0.37	0.39	0.4	0.41

**Table 5:** Forecast of coupling coordinative degree among the three systems.

Cities	2018	2019	2020	2021	2022
Jinan	0.6972				
Qingdao	0.8359	0.8692	0.9039	0.9399	0.9774
Zibo	0.6038	0.6277	0.6525	0.6784	0.7052
Zaozhuang	0.4615	0.4842	0.5081	0.5330	0.5593
Dongying	0.4696	0.4835	0.4978	0.5126	0.5277
Yantai	0.7532	0.7697	0.7865	0.8037	0.8213
Weifang	0.5993	0.6187	0.6388	0.6596	0.6810
Jining	0.5583	0.5684	0.5787	0.5892	0.5999
Taian	0.5211	0.5361	0.5517	0.5676	0.5840
Weihai	0.5497	0.5655	0.5818	0.5986	0.6159
Rizhao	0.4770	0.4940	0.5117	0.5299	0.5488
Laiwu	0.3589				
Linyi	0.5912	0.6103	0.6301	0.6506	0.6717
Dezhou	0.4755	0.4890	0.5029	0.5172	0.5319
Liaocheng	0.4994	0.5315	0.5656	0.6019	0.6405
Binzhou	0.4134	0.4228	0.4323	0.4420	0.4520
Heze	0.4321	0.4501	0.4687	0.4882	0.5084

## Data Availability

The data presented in this study are available on request from the corresponding author. The data are not publicly available due to data publisher regulations.
